# High-density evaluation of the arrhythmogenic substrate in persistent atrial fibrillation

**DOI:** 10.1016/j.hroo.2025.06.019

**Published:** 2025-07-01

**Authors:** Haseeb Valli, Mahmoud Ehnesh, Sam Coveney, David G. Jones, Zhong Chen, Wajid Hussain, Vias Markides, Kumaraswamy Nanthakumar, Tom Wong, Caroline Roney, Shouvik Haldar

**Affiliations:** 1 Heart Rhythm Centre, Royal Brompton & Harefield Hospitals, Guy’s and St Thomas’ NHS Foundation Trust, Hill End Road, Harefield, Uxbridge, United Kingdom; 2 National Heart and Lung Institute, Imperial College London, Cale Street, London SW3 6LY, UK; 3 School of Engineering and Materials Science, Queen Mary University of London, London, United Kingdom; 4 Leeds Institute of Cardiovascular and Metabolic Medicine, University of Leeds, Leeds, United Kingdom; 5 Hull Family Cardiac Fibrillation Management Laboratory, Division of Cardiology, Department of Medicine, University of Toronto, Toronto, Ontario, Canada

**Keywords:** Atrial fibrillation, Ablation, Substrate, Voltage, Omnipolar

## Abstract

**Background:**

Left atrial (LA) fibrosis is a key component of arrhythmogenic remodeling in atrial fibrillation (AF). LA low-voltage areas (LVAs) are considered surrogates for fibrosis and novel targets for ablation. However, there are no established criteria for identifying such potential pathogenic areas, particularly when using omnipolar technology (OT) mapping.

**Objective:**

This study aimed to evaluate the correlation between OT and conventional bipolar voltage (BiV) in AF and regular rhythms.

**Methods:**

Bipolar and OT mapping was performed in 17 patients undergoing de novo ablation for persistent AF. Mapping was performed in AF and coronary sinus pacing (CSP) at 600 ms. BiV of <0.5 mV was defined as low voltage.

**Results:**

LA voltage in AF correlated poorly with CSP using either BiV (r = 0.15) or OT (r = 0.16). OT yielded higher voltages than BiV in AF (0.62 ± 0.24 vs 0.49 ± 0.18 mV, *P* < .050) and during CSP (1.85 ± 0.78 vs 1.60 ± 0.80 mV, *P* < .050). LVA burden, as a percentage of LA surface area, varied significantly depending on the atrial rhythm and mapping approach (AF-bipolar 65.0 ± 15.6%, AF-OT 56.2 ± 17.0%, CSP-bipolar 34.2 ± 18.9%, CSP-OT 24.56 ± 13.5%, *P* < .050). BiV thresholds of 0.5 mV during CSP and 0.3 mV in AF corresponded to an OT voltage of 0.84 mV and 0.40 mV, respectively.

**Conclusion:**

The mapping tool and atrial rhythm significantly influence LA voltage and LVA burden for both bipolar and OT mapping. Applying a universal bipolar or OT cutoff for low voltage in AF and sinus rhythm will not accurately reflect the arrhythmogenic substrate. OT yields higher voltage than corresponding bipolar measurements; thus, threshold adjustments are required when using OT.

## Graphical abstract

**Figure:**
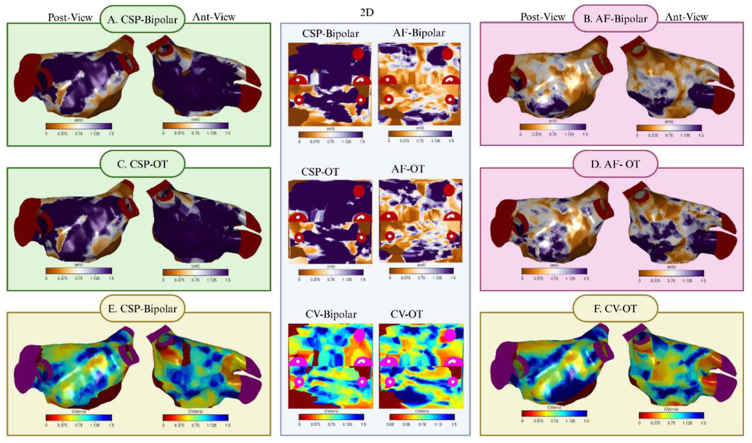

**Key Findings**

▪ Left atrial (LA) low-voltage areas (LVAs), considered to represent regions of arrhythmogenic fibrosis, increase the risk of atrial fibrillation (AF) recurrence after ablation.
▪ Adjunctive ablation targeting LVAs is associated with improved freedom from AF.
▪ LA voltage and burden of LVAs are highly dependent on the atrial rhythm during mapping and the mapping approach used.
▪ Omnipolar technology (OT) yields higher voltages and in turn smaller LVAs than conventional bipolar voltage (BiV); however, there remains a significant difference between AF and regular rhythms.
▪ BiV thresholds of 0.5 mV during coronary sinus pacing and 0.3 mV in AF correspond to a voltage of 0.84 mV and 0.40 mV respectively, when OT is used.

## Introduction

Catheter ablation to eliminate pulmonary vein (PV) triggers is highly effective in maintaining sinus rhythm (SR) in certain patients with atrial fibrillation (AF). However, arrhythmia recurrence is observed in a sizable proportion of patients with paroxysmal AF (PAF) and more than half of those with nonparoxysmal forms,^1^ suggesting that sites beyond the PVs are also involved in AF pathogenesis. Adjunctive ablation to modify the atrial arrhythmogenic substrate has been attempted, but no approach has shown improved outcomes in randomized controlled trials (RCTs).^2^

Recently, substrate modification targeting regions of suspected atrial fibrosis has garnered increasing enthusiasm.^3^ Progressive fibrosis is a key component of adverse remodeling associated with AF persistence.^4^ The burden of fibrosis, measured as atrial late gadolinium enhancement (LGE) on cardiac magnetic resonance (CMR) imaging, correlates with AF recurrence after ablation.^5^ Atrial low-voltage areas (LVAs) seen on electroanatomic mapping (EAM), considered a surrogate for fibrosis, similarly predict such recurrence.^6^ However, ablation targeting sites matching the LGE distribution on CMR imaging was not superior to PV isolation (PVI) in maintaining SR.^7^ Substrate modification targeting LVAs has shown encouraging results in early observational studies. However, outcomes from more recent RCTs have been less consistent.^8^

These inconsistent outcomes from recent LVA ablation studies highlight important limitations in identifying sites critical to arrhythmogenesis. Voltage assessment is influenced by many factors, including atrial rhythm and mapping catheter properties, with no consensus on the optimum strategy for identifying LVAs. Omnipolar technology (OT) overcomes several limitations of conventional bipolar voltage (BiV) assessment, potentially providing a more accurate representation of left atrial (LA) voltage and improving the delineation of LVA.^9^ However, there are limited data on how LVAs are defined when using OT, and the mechanistic role of LVAs in AF remains unclear. The present study compared LA voltage measurements from conventional BiV with those obtained using novel OT. We posited that OT, by capturing direction-independent voltage, could better characterize the atrial substrate in AF, particularly in regions with anisotropic conduction where preferential pathways may exist despite the chaotic nature of AF. Voltage comparisons were undertaken in AF and during regular rhythms and compared with other electrophysiological parameters, such as conduction velocity (CV), to clarify the pathologic significance of LVAs.

## Methods

### Study population

This was a single-center prospective study at the Royal Brompton & Harefield Hospitals, enrolling patients with persistent AF (PsAF) of <18-month duration. Patients referred for catheter ablation were eligible for the study if they were ≥18 and ≤80 years old and had symptomatic AF refractory to antiarrhythmic drugs (AADs). Key exclusion criteria included previous LA ablation, significant coronary artery or valvular heart disease, contraindication to anticoagulation, presence of a cardiac implantable electronic device, and cerebrovascular accident within 6 months. The study protocol complies with the Declaration of Helsinki. All patients provided a written consent for their study participation, which the UK National Research Ethics Service approved.

### Electrophysiological mapping and ablation

AADs were discontinued 5 days prior to the procedure, and amiodarone was stopped 6 weeks before ablation. All procedures were performed under general anesthesia on uninterrupted anticoagulation. A decapolar catheter was introduced through the coronary sinus, and transseptal access to the LA was obtained using standard techniques. Intravenous heparin was administered to maintain an activated clotting time of >300 seconds.

EAM was performed with the EnSite X using the Advisor HD grid mapping catheter with a 4 × 4 arrangement of electrodes (1 mm size, 3 mm spacing) (Abbott Medical Inc, St. Paul, MN). Detailed LA voltage maps were recorded in all patients. For patients presenting in AF at the start of the procedure, voltage maps were first recorded in AF, followed by external direct current (DC) cardioversion to SR. Repeat voltage mapping was performed with proximal coronary sinus pacing (CSP) at 600 ms.

BiV maps were produced from the peak-to-peak voltage with amplitudes of >0.5 mV considered normal; amplitudes of <0.5 mV were defined as low voltage. OT voltages were obtained from a triangular clique of 3 nonlinear adjacent electrodes and localized to the center of the clique.

PVI was thereafter performed using the TactiFlex ablation catheter (Abbott Medical Inc), with bidirectional conduction block serving as the endpoint. Electrogram (EGM) and CV analysis were performed using custom scripts in MATLAB (MathWorks, Natick, MA); a detailed description is provided in the Supplemental Data and Supplemental Figures 1 and 2.

### Statistical analysis

Statistical analyses were conducted using GraphPad Prism (GraphPad Software, Inc, San Diego, CA). Continuous variables are presented as means ± standard deviations, whereas categorical variables are expressed as numbers and percentages. The Student’s *t* test was applied to compare continuous variables. To compare the point-by-point recordings with the high-density Gaussian process manifold interpolation (GPMI) maps, normality was first assessed using the Shapiro-Wilk test (α = 0.05). A paired-sample *t* test was then conducted to evaluate differences in correlation values between the 2 methods. *P* < .050 was considered statistically significant.

## Results

### Patient characteristics

A summary of baseline patient characteristics is presented in Table 1. The mean age was 67.4 ± 5.2 years, and 88% were men. Left ventricular function was preserved (mean 61.18 ± 5.22%), and there was evidence of mild LA dilatation (mean area indexed to height 14.24 ± 2.07 cm). Notably, 94% were on AAD at the time of ablation, and 5 patients had a history of treatment with amiodarone.

**Table 1:** Clinical characteristics of study population

Category	Total Population (n = 17)
Sex (M/F)	2/15
Age (y)	67.41 ± 5.37
BMI (kg/m^2^)	28.82 ± 4.84
AF duration (mo)	17.12 ± 2.61
LA size (indexed surface area)	14.24 ± 2.07
LV function (%)	61.18 ± 5.22
Previous antiarrhythmic drug (n)	
Class I	1
Class II	17
Class III	2
Class IV	5
Hypertension (n)	14
Diabetes mellitus (n)	1
Vascular disease (n)	3
CHADS2-VASc	2.06 ± 1.10
Previous DCCV (n)	16

Table showing baseline characteristics of patients. Continuous variables are given as mean ± standard deviation.

AF = atrial fibrillation; BMI = body mass index; DCCV = direct current cardioversion; F = female; LA = left atrium; LV = left ventricle; M = male.

### Assessment of mean voltage

Ten patients presented in AF, so EAM was undertaken prior to DC cardioversion and CSP mapping. Seven patients were in SR at the onset of the procedure, and only CSP data were collected.

After exclusion of the PVs and LA appendage, a total of 49,913 bipolar and 67,098 OT EGMs were analyzed during CSP (mean 2936 ± 805 bipolar and 3947 ± 1025 OT EGMs per LA). Similarly, 26,382 bipolar and 35,508 OT EGMs were collected in AF (mean 2638 ± 925 bipolar and 3550 ± 1392 OT EGMs per LA).

Mean peak-to-peak voltage was significantly higher during CSP than AF for bipolar (1.60 ± 0.80 vs 0.49 ± 0.18 mV, *P* < .001) and OT EGMs (1.85 ± 0.78 vs 0.62 ± 0.24 mV, *P* < .001). OT yielded higher voltage than BiV across all rhythms (CSP *P* < .050, AF *P* < .001). Accordingly, the burden of LA LVAs as a percentage of total surface area, when applying a uniform voltage threshold to define low voltage, is lower during CSP than AF and when using OT than BiV (Table 2).

**Table 2:** Low voltage area burden according to mapping rhythm and mapping approach

Rhythm	Voltage mapping modality	
	Bipolar	Omnipolar
AF	65.00 ± 15.6	56.20 ± 17.0
CSP	34.13 ± 18.9	24.56 ± 13.5

Low-voltage area burden given as a percentage of the total left atrial surface area according to mapping rhythm (AF and CSP) and mapping approach.

AF = atrial fibrillation; CSP = coronary sinus pacing.

### Correlation between mapping techniques

**Figure 1:**
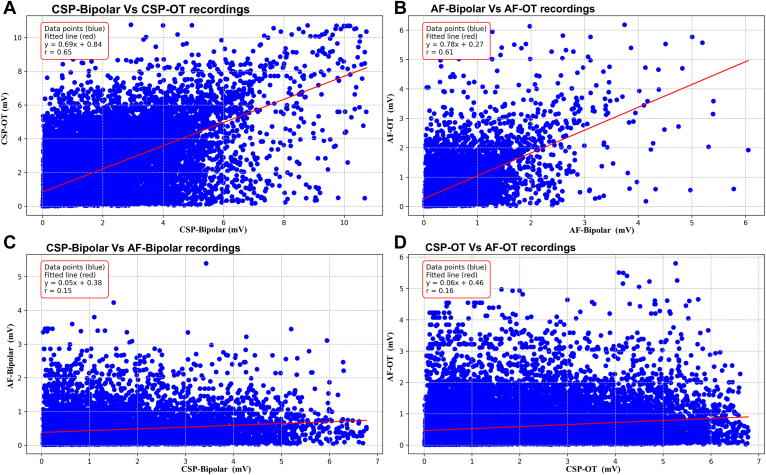
Comparative analysis of EGM mapping modalities during different rhythm conditions. A: Comparisons between bipolar and OT recordings during CSP. B: Similar comparisons during AF. All data points are included, before excluding outliers as per the exclusion criteria described in the Methods. C: Comparisons between the same EGM recordings across different rhythms: CSP-bipolar vs AF-bipolar. Similarly, panel D compares CSP-OT with AF-OT recordings. AF = atrial fibrillation; CSP = coronary sinus pacing; EGM = electrogram; OT = omnipolar technology.

Correlation between peak-to-peak voltage values obtained under varying mapping conditions was performed by pairing points according to location on the LA surface (Figure 1). There was a good correlation between bipolar and OT voltages during CSP (r = 0.65) and AF (r = 0.61). In contrast, BiVs in AF correlated poorly with those recorded during CSP (r = 0.15). OT voltages in AF were higher than those measured with BiV across the observed voltage range. However, the correlation between CSP and AF with OT was weak (r = 0.16).

**Figure 2:**
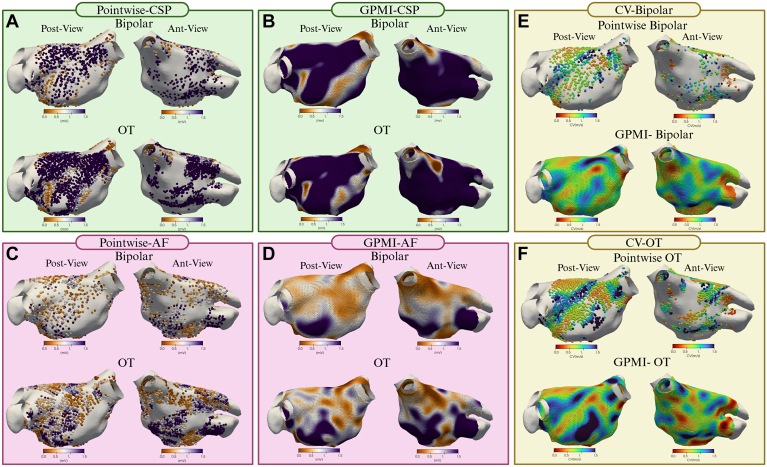
Comparative analysis of peak-to-peak voltage recordings and GPMI method maps. **A:** Posterior and anterior wall views of peak-to-peak voltage recordings from bipolar and OT maps CSP. **B:** Pointwise maps of peak-to-peak voltage recordings interpolated using the GPMI method. **C and D:** Voltage mapping during AF with peak-to-peak voltage recordings (**C**) and GPMI interpolation (**D**). **E and F:** CV maps of the LA during CSP, with BiV (**E**) and OT (**F**). AF = atrial fibrillation; Ant = anterior; BiV = bipolar voltage; CSP **=** coronary sinus pacing; CV = conduction velocity; GPMI = Gaussian process manifold interpolation; OT **=** omnipolar technology; Post = posterior.

**Figure 3:**
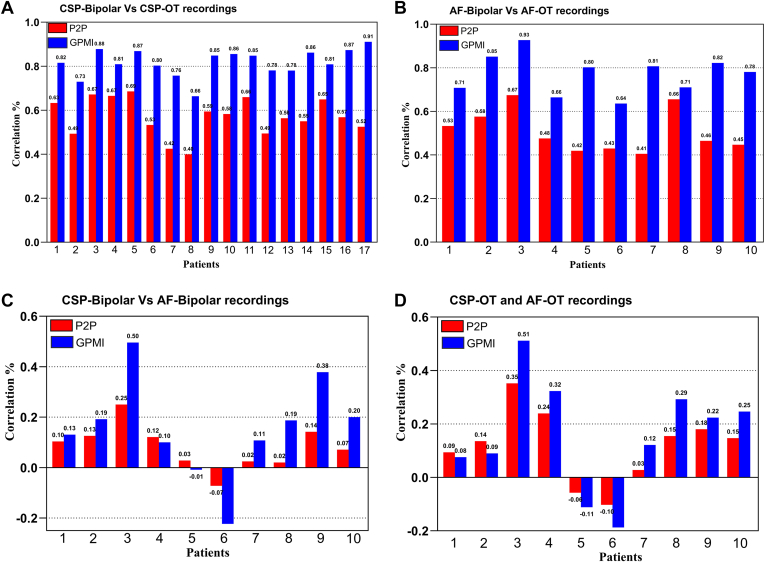
Pointwise comparison of bipolar and OT during CSP and AF. The colors represent correlation values, with *red* indicating peak-to-peak voltage measurements and *blue* representing the GPMI method. (A) Correlation between CSP-bipolar and CSP-OT recordings. (B) Correlation between AF-bipolar and AF-OT recordings. (C) Correlation between CSP-bipolar and AF-bipolar recordings. (D) Correlation between CSP-OT and AF-OT recordings. AF = atrial fibrillation; CSP = coronary sinus pacing; GPMI = Gaussian process manifold interpolation; OT **=** omnipolar technology.

To further investigate the correlation between voltages in AF and CSP, we applied novel GPMI analyses to improve the spatial resolution and accuracy of the voltage maps (Figure 2).^10^^,^^11^ Using the GPMI analysis, voltages measured with conventional BiV correlated strongly with those seen with OT during CSP and AF (Figure 3A and 3B). Although correlation coefficients were higher with the GPMI method, the overall correlation between CSP and AF remained weak for both bipolar and OT mapping (Figure 3C and 3D).

### Regional analysis of voltage

**Figure 4:**
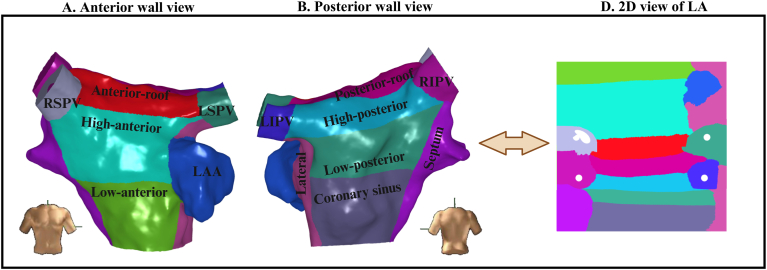
Regional segmentation of the left atrium (LA). The LA was segmented into 14 predefined regions, including the 4 pulmonary veins, the mitral valve (MV) region, the coronary sinus (CS) region, the LAA, the anterior and posterior walls (further divided into superior and inferior portions), the lateral wall, the roof, the inferior region, and the septum. A shows the anterior view, B shows the posterior view, and D displays the 2D view. 2D = 2-dimensional; LAA = left atrial appendage; LIPV = left inferior pulmonary vein; RSPV = right superior pulmonary vein.

**Figure 5:**
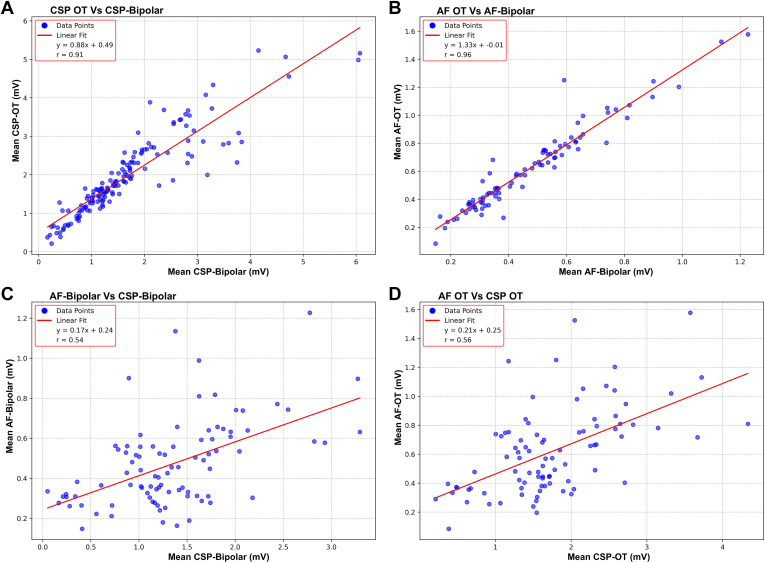
Regional analysis of the relationship between bipolar and OT recordings during CSP and AF. Each plot presents the results of linear regression analysis comparing the mean voltage in specific anatomic regions of the LA across 14 predefined regions for each modality. **A:** Linear regression of CSP-OT vs CSP-bipolar recordings. **B:** Linear regression of AF-OT vs AF-bipolar recordings. **C:** Linear regression of AF-bipolar vs CSP-bipolar recordings. **D:** Linear regression of AF-OT vs CSP-OT recordings. AF = atrial fibrillation; CSP = coronary sinus pacing; LA =left atrium; OT **=** omnipolar technology.

LA voltage has been shown to vary according to location. To evaluate potential regional variations in LA voltage, the LA was segmented into 14 regions, as shown in Figure 4. Mean voltage was calculated for each region in AF and during CSP, using bipolar and OT mapping. Such averaging across predefined anatomic regions obviates the limitations of accurately pairing individual points between maps. There was a very strong correlation between regional mean BiV and mean voltage obtained with OT during CSP (r = 0.91) and AF (r = 0.96) (Figure 5A and 5B). Mean regional voltage was higher during CSP than in AF; however, in contrast to the point-by-point analysis, there was a moderate correlation between AF and CSP voltages with either BiV (r = 0.54) or OT (r = 0.56) (Figure 5C and 5D).

### CV analysis

LA LVAs have been proposed as sites important for AF maintenance. Therefore, we evaluated the potential for conduction slowing as a mechanism underlying the arrhythmogenic potential.^12^ As shown in Supplemental Figure 3A, LA bipolar and OT voltages during CSP correlated poorly with CV measurements for all included patients. When bipolar and OT voltages in AF were compared with the corresponding CV, no correlation was evident. When assessed on a regional basis, there was a modest correlation between CSP voltage and CV with bipolar mapping (r = 0.41), but only weakly for OT (r = 0.22) (Supplemental Figure 3B). Correlations between regional mean voltage in AF and CV were poor for both bipolar (r = 0.21) and OT mapping (r = 0.17).

### Voltage threshold analysis

**Figure 6:**
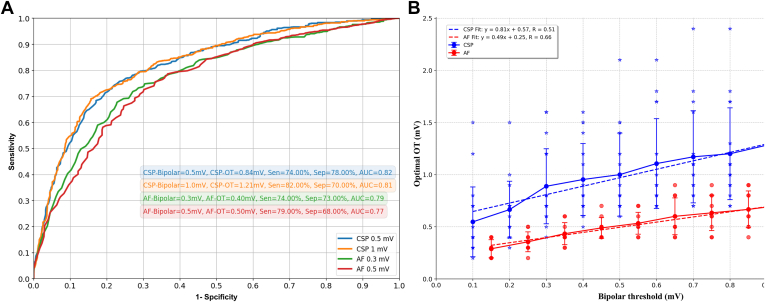
ROC curves illustrate the identification of low-voltage areas (LVAs) for each rhythm and bipolar threshold. **A:** Each line represents the sensitivity and specificity across varying OT thresholds, based on 4 distinct BiV thresholds reported in the literature for CSP and AF. The median optimal OT threshold, calculated across all patients for each bipolar threshold, is provided. **B:** Relationship between bipolar and OT voltage thresholds in CSP (*blue*) and AF (*red*). The OT threshold with the highest concordance to the bipolar map is shown for various bipolar thresholds across individual patients (*blue stars* and *red dots* represent CSP and AF, respectively). Means are indicated as continuous lines, and standard deviations are indicated by error bars. The optimal OT threshold is determined as the point on the ROC curve corresponding to each bipolar threshold across all patients. Linear regression lines are depicted (dotted), with Pearson correlation coefficients (r) provided for CSP and AF. AF = atrial fibrillation; AUC = area under the curve; BiV = bipolar voltage; CSP = coronary sinus pacing; OT **=** omnipolar technology; ROC = receiver operating characteristic; Sen = sensitivity; Sep = specificity.

Given that OT yielded higher peak-to-peak voltage than conventional BiV, we sought to define optimal OT thresholds that would correspond to commonly used BiV thresholds. A receiver operating characteristic (ROC) analysis for OT voltages corresponding to low- and high-voltage thresholds with BiV in AF and during CSP is presented in Figure 6A. A BiV threshold of 0.5 mV corresponded to a voltage of 0.84 mV using OT (sensitivity 74%, specificity 78%, area under the curve 0.82). For mapping in AF, a bipolar threshold of 0.3 mV was equivalent to an OT voltage of 0.40 mV (sensitivity 70%, specificity 74%, area under the curve 0.79).

ROC analyses were performed for BiV thresholds from 0.1 mV to 1 mV in 0.1 mV increments to identify the OT voltage threshold for each bipolar value. The results of the linear regression analysis for the threshold values are shown in Figure 6B. The results yielded a moderate positive correlation for both AF (r = 0.66) and CSP (r = 0.51).

## Discussion

This study presents a highly detailed evaluation of voltage-guided atrial substrate assessment, using novel OT in patients undergoing first-time ablation for PsAF. The principal findings of the study are as follows:

1) LA voltage and the burden of LA LVAs are significantly influenced by the mapping strategy used.
2) LA voltage recorded in AF correlates poorly with those measured during regular CSP, for both bipolar and omnipolar mapping.
3) OT yields significantly higher voltage measurements than conventional bipolar mapping.
4) Optimal OT voltage values corresponding to commonly used thresholds for delineating LVAs were identified.

### Voltage differences in AF and regular pacing

In the present study, voltage assessment was undertaken with fast anatomic mapping using a high-density multipolar catheter, analyzing approximately 50,000 EGMs during CSP and more than 65,000 in AF, with an average of >2500 points per map. We observed a 3-fold reduction in mean LA voltage in AF compared with regular CSP. Moreover, voltage measurements in AF correlated very poorly with those observed during CSP. Ndrepepa et al^13^ have previously reported a very similar magnitude difference in LA voltage between AF and SR. Yagishita et al^14^ also reported lower voltages in AF than in SR, but found a linear correlation between the rhythms (r = 0.707), although mapping was performed with a 3.5 mm bipolar catheter resulting in low-point density maps (approximately 100 points per map). A more recent study with multipolar mapping analyzing a modest total of 2002 points across all maps combined reported a moderate correlation between AF and SR (Kendall’s tau = 0.56).^15^

Accurate pairing of voltage measurements between maps can be challenging owing to myocardial contraction through the cardiac cycle, respiratory motion, and chamber distortion through catheter contact. Some of these variables can be improved through proprietary respiratory gating algorithms and setting windows of interest for data collection. We first compared AF voltage with CSP voltage in a point-by-point fashion by pairing points based on location, with a very poor correlation between the rhythms. We also used the novel GPMI method to improve the accuracy of interpolation and spatial resolution of the maps.^10^ Although the correlation between maps generally improved with GPMI analysis,^11^ the correlation between AF and CSP remained poor. Finally, the regional mean voltage was compared by segmenting the LA into predefined anatomic regions. Comparing regional voltages improved correlations across all analyses, presumably through summating heterogeneity in individual points within a region. However, the correlation between AF and CSP was moderate at most.

We found higher correlation coefficients in points with more normal voltage in AF, whereas points with low voltage in AF correlated particularly poorly to corresponding points during CSP. In keeping with this, Teh et al^16^ reported significantly higher CSP voltages than in AF and observed no correlation in LA voltages between CSP and AF. Moreover, regions of LVA and complex fractionated atrial EGMs observed in AF seemed ostensibly normal when assessed during the paced rhythm. Masuda et al^17^ reported a good correlation between SR and AF voltages (r = 0.73) in areas where EGM morphology was normal in AF. However, regions displaying normal EGMs in SR frequently exhibited fractionation in AF, with poor correlation in BiVs at such sites. Thus, the pathologic significance of LVA and EGM fractionation in AF remains unclear and may not be an appropriate target for substrate modification in AF. The differences in LA voltage and LVA burden between AF and CSP, in part, reflect differences in atrial activation rates. Repeating such measurements at different atrial pacing rates would help further elucidate the rate dependence of these parameters. Interestingly, Wong et al^18^ reported significant variations in LA voltage, burden of LVAs, and EGM fractionation during atrial pacing, depending on the pacing site and atrial rate.

### OT

OT has been reported to overcome technical limitations associated with conventional bipolar EGMs, obviating issues with electrode orientation and activation timing.^9^^,^^19^, ^20^, ^21^ In animal studies, OT voltages demonstrated remarkable beat-to-beat consistency across rhythms, potentially reducing the influence of rhythms with variable activation patterns such as AF.^22^ OT voltages correlated strongly with corresponding BiV measurements in AF and during CSP. Overall, the mean OT voltage was significantly higher than BiV for the same rhythm, and accordingly, the burden of LVAs was smaller with OT than BiV. However, like BiV measurements, the overall mean OT voltage during CSP was higher than in AF, and OT AF voltage correlated weakly with that measured during CSP.

Similar to this study, Rillo et al^23^ reported higher atrial voltages and a smaller burden of LVAs in patients undergoing AF ablation when mapped in SR. Butcher et al^24^ reported that OT voltage maps in AF better correlated with BiV maps in SR than bipolar AF voltages in 20 patients undergoing first-time ablation for PsAF. The mean BiV in SR was relatively low at 0.62 mV, and the absolute difference between bipolar and OT voltage in AF was of a similar magnitude to that seen in the present study. It is yet to be determined whether OT mapping in AF can bridge the gap between AF and SR in a larger cohort of patients with varying substrates. Yavin et al^25^ reported limited utility of OT mapping compared with BiV in the real-world setting of roving catheters, where direction independence may be less relevant. Our derivation of an OT threshold equivalent to the BiV standard provides a novel reference for future studies, although validation in larger cohorts is necessary to establish clinical utility.

### LA LVAs and AF

Adverse atrial electrical and structural remodeling underpins AF persistence and its resistance to treatment, with atrial fibrosis forming an important component of the latter. Atrial fibrotic remodeling has been associated with AF in postmortem analyses,^26^ and the extent of fibrosis correlates with AF duration.^27^ Atrial LGE seen on CMR imaging has been used to identify myocardial fibrosis, and the degree of LGE has been shown to predict recurrent AF after ablation.^28^ These reports have prompted interest in identifying and ablating fibrotic regions within the LA.

LA LVAs have been suggested to represent regions of atrial fibrosis and thus serve as novel targets for ablation. A higher prevalence of LVAs has been reported in patients with nonparoxysmal AF than in those with paroxysmal AF.^14^ Early observational studies demonstrated improved freedom from AF with targeted ablation of LVAs as an adjunct to PVI.^3^ However, outcomes from more recent RCTs have been variable,^8^^,^^29^ highlighting the need for further refinements to this approach.

When evaluating such LVA ablation studies, studies vary significantly with respect to catheter properties, thresholds for delineating pathologically low-voltage regions, and atrial rhythm during mapping. These factors, along with a host of others, contribute to the measured peak-to-peak voltage, and the absence of standardization is likely to have contributed to the mixed outcomes reported in these studies.

The precise role of atrial LVAs in initiating and/or perpetuating AF remains unclear. In patients with PsAF, high dominant frequency sites are often colocated with LVAs or their border zones.^30^ LA LVAs have also been reported to frequently harbor complex fractionated atrial EGMs.^31^ Interestingly, some studies have reported conduction slowing in areas with a BiV of <0.5 mV.^32^ In contrast, we did not observe conduction slowing in regions of low bipolar or OT voltage, either in AF or CSP (Supplemental Data).

Moreover, the overall LVA burden during CSP was very low in our study cohort of PsAF. This is consistent with other studies of LVA ablation, where LA LVAs are identified in less than half of the study populations.^3^ The absence of LVAs in many patients with nonparoxysmal AF raises questions about the accuracy of voltage assessment in identifying arrhythmogenic atrial remodeling. Given this observation and the absence of demonstrable conduction slowing in the modern era of high-resolution multipolar mapping, there may be a need to re-evaluate the voltage cutoff used to highlight tissue with arrhythmogenic potential. Nonetheless, a recent meta-analysis of RCTs investigating outcomes from LVA ablation has suggested improved freedom from AF in patients with PsAF but not PAF, suggesting that adopting this strategy may improve outcomes overall.^33^ However, our findings suggest particular uncertainty regarding the clinical significance of LVAs identified during mapping in AF, and perhaps such evaluation should be performed in regular rhythms such as CSP. To further address the potential utility of targeted ablation of LVAs detected with different mapping approaches and rhythms, we are conducting a parallel computational modeling study to simulate ablation outcomes based on LVA characteristics.

### Optimal voltage thresholds

The mapping approach used significantly influences the measured voltage and must be considered when interpreting voltage maps and deciding upon ablation strategies. In our analyses, a BiV of 0.5 mV in CSP corresponded to an OT voltage of 0.84 mV. Similarly, a BiV of 0.3 mV in AF corresponded to an OT voltage of 0.4 mV. Given the poor correlation between AF and CSP for both bipolar and OT mapping, we could not propose voltage thresholds to translate AF and CSP maps. Other studies have suggested a stronger correlation between AF and regular rhythms. For example, Rodríguez-Mañero et al^15^ suggested that a voltage threshold of 0.5 mV in SR would equate to a voltage of 0.25 mV in AF and 0.35 mV in atrial flutter.

### Future directions

High-density substrate characterization using contemporary multielectrode mapping tools in patients with PsAF yields less LA LVA burden than anticipated. This suggests a hidden substrate within healthy voltage areas outside of the PV, which may require additional dynamic functional testing to unmask. Such extrastimulus testing has emerged as a useful tool to highlight proarrhythmic functional changes in ventricular arrhythmias. Frontera et al^34^ recently reported areas of conduction slowing within the LA identified during dynamic pacing. The presence of such sites predicted AF recurrence after PVI, and of note, most of these sites were remote from regions of low voltage. Further studies are needed to investigate the utility of dynamic functional testing and the ablation of putative arrhythmogenic sites.

### Limitations

This was a small single-center study in a relatively homogeneous group of patients and may not apply to all patients with AF. However, we collected a more significant number of mapping points than previous similar studies. Nonetheless, given the small sample size, the 0.84 mV OT threshold requires validation. Further insights from computational modeling may add credence to the thresholds proposed in our analyses. In patients presenting in AF, DC cardioversion was performed after collecting AF mapping data. This may have resulted in subtle shifts in geometry beyond the sensitivity of the mapping system. In addition, CSP data were collected shortly after the restoration of SR. It is known that some aspects of atrial remodeling do reverse over the long term if SR is maintained. Therefore, it is difficult to ascertain how representative the acute CSP map is of the atrial electrophysiological parameters in SR. In addition, we have not explored the influence of alternative catheters or mapping software on the data obtained. The study did not investigate the reproducibility of voltage measurements and LVA burdens across different AF episodes using either BiV or OT mapping, which could inform the stability of identified substrates. Future studies should explore this to validate the consistency of LVA identification. The CV calculations may be affected by fractionated EGMs in fibrotic regions, although we mitigated this by using EnSite X’s local activation time assignment.

## Conclusion

LA voltage is highly dependent on the atrial rhythm during mapping and the recording strategy used. Thus, LVA burden and distribution will also vary accordingly. In the current study, we demonstrate that although OT yields higher voltage than bipolar mapping, there remains a significant voltage difference when mapping in AF and regular rhythms. Moreover, we did not observe arrhythmogenic conduction slowing in LVAs. Therefore, although the presence of LVAs serves as an adverse prognostic marker in AF from a rhythm control perspective, the pathologic significance remains unclear and might explain the inconsistent outcomes from LVA ablation studies.
