# Supplementary material for: High-density evaluation of the arrhythmogenic substrate in persistent atrial fibrillation
Source: Heart Rhythm O2. 2025 Jul 1;6(10):1536–45. doi: 10.1016/j.hroo.2025.06.019 (PMC12570181; doi:10.1016/j.hroo.2025.06.019)
Supplement: Supplementary Data [file mmc1.docx]

**Supplemental methods:**

**Voltage Mapping**

Bipolar voltage maps were produced from the peak-to-peak voltage measured from bipoles along the spline of the HD-grid catheter. All electrograms (EGMs) were filtered at 30 and 500Hz. Maximum peak-to-peak voltage was identified from all atrial EGMs falling within the window of interest between two consecutive QRS complexes using the best duplicate algorithm. EGMs 5mm from the geometry surface were automatically excluded, and an interpolation threshold of 7mm was used. Bipolar voltage amplitudes >0.5mV were considered normal; amplitudes <0.5mV were defined as low voltage and <0.05mV as very low voltage. OT voltages were obtained from a triangular clique of three non-linear adjacent electrodes and localised to the centre of the triangular clique.

Following adequate geometry and voltage map acquisition, PVI was performed using the Tactiflex ablation catheter (Abbott Medical Inc., MN). Ablation was performed at 35-50w according to operator discretion with a minimum contact force of 5g and bidirectional conduction block serving as the endpoint. Finally, cavotricuspid isthmus ablation was performed in patients with documented typical atrial flutter.

**Electrogram Analysis**

Point-by-point analyses were performed following the removal of extraneous recordings. Outliers were removed by applying a mean ± 2 SD of the CSP bipolar recordings as a reference standard, maintaining the upper voltage limit within the physiological range for all patients. An individual LA mesh was created for each patient. The pre-processing workflow involved surface smoothing and re-meshing of all LA meshes using Meshmixer, followed by removing extra structures (e.g., PVs and mitral valve) via clipping in ParaView. To further refine the mesh, Laplacian smoothing, element subdivision, and short-edge collapsing were applied using MeshLab, ensuring a regular mesh topology and eliminating self-intersections. These steps improved mesh quality, enabling accurate assignment of EGM data to the nearest vertices on the processed mesh^1^. A mean+2 standard deviation (SD) threshold was applied to the peak-to-peak CSP voltage to exclude outlier EGM recordings and used as a reference. A sample of EGM recordings from four patients is illustrated in supplemental figure 1A. The closest point-matching technique was employed for spatial registration of EGMs from different mapping rhythms. This algorithm spatially aligned corresponding EGM locations between bipolar and OT recordings, ensuring that each bipolar EGM was paired with its nearest OT counterpart. A 5mm cut-off distance threshold was applied for matched points, enabling a robust evaluation of voltage, signal characteristics, and correlation differences between bipolar and OT recordings collected during different rhythms, an example for one patient of the point-matching technique, in which the EGMs at different rhythms were paired with each other, within the threshold distances of 5 mm shown in supplemental figure 1B. The percentage of LVAs within the LA was determined using a predefined threshold of 0.5mV. For each patient, voltage recordings below the threshold were identified and separated from those with values above the threshold. The percentage of the LVA was computed relative to the total voltage surface area.

A major challenge in quantifying EGM recordings is accurately registering and interpolating points on complex atrial geometry. The Gaussian Process Manifold Interpolation (GPMI) method, as proposed by Coveney et al.^2^, offers a substantial improvement over traditional interpolation techniques by providing a probabilistic framework for interpolating uncertain EGMs recordings directly onto the complex geometry of the atrial surface. This method enables comprehensive coverage of the atrial surface by interpolating sparse EGM recordings while simultaneously quantifying the uncertainty in the recordings. By applying this probabilistic model, mean values and standard deviations of the interpolated EGMs are obtained, and the mean values are then used to replace low-resolution EGM recordings and assign values to all vertices on the anatomical mesh. This approach, referred to as GPMI, not only enhances the spatial resolution but also provides a statistical measure of confidence in the interpolated values, making it a powerful tool for improving the accuracy of atrial electrophysiology mapping^1^,^3^. Thus voltage comparisons were undertaken with conventional pointwise and GPMI approaches.

CV was computed exclusively during CSP using cosine wavefront-fitting methods, with a fitting threshold applied to exclude regions with multiple wavefronts or collisions. This technique estimates CV by assuming either a planar or circular wave propagating uniformly across recording sites. The approach is particularly suitable for clinically acquired electrophysiological data with varying catheter configurations. Specifically, CV was calculated by estimating local gradients from the local activation time (LAT) field, fitting a planar wavefront to LAT measurements within a 6-mm radius around each recording location^1^,^3^. This method helps mitigate errors introduced by non-uniform electrode distributions and ensures a physiologically relevant interpretation of wavefront propagation. Also, by adapting to the local activation pattern, the technique enables the characterisation of conduction heterogeneities, which is essential for understanding arrhythmogenic substrates and abnormal conduction pathways.

For regional analysis, the LA was subdivided into fourteen anatomical regions. The universal atrial coordinate (UAC) system was employed to transfer these region labels to the meshes of other patients, ensuring consistent regional segmentation across all geometries.

**Statistical Analysis**

To evaluate the correlation between bipolar and OT voltage maps, sensitivity and specificity were calculated, with the bipolar map serving as the reference standard. Each EGM point in the OT map was classified based on an OT voltage threshold, using the bipolar map as the ground truth. Points classified as low voltage in the gold standard bipolar map were labelled as true positives (OT voltage < threshold) or false negatives (OT voltage > threshold) in the OT map. Conversely, true negatives and false positives were assigned to points with above-threshold voltage in the bipolar map. The OT voltage threshold was varied between 0.1 and 6 mV in 0.1 mV increments (as 6 mV represents the OT voltage range for all patients), and a receiver operating characteristic (ROC) curve was generated to determine the OT threshold that best aligned with the bipolar map for each patient and rhythm.

**Supplemental results**

**Correlation between mapping techniques**

Previous studies have suggested correlation in LA voltage across different rhythms varies as a function of voltage amplitude^4^. To assess such a relationship between recorded voltage and correlation between maps, mapping points were segregated into voltage ranges, and their respective correlation coefficients were compared (supplemental figure 2). In all comparisons, correlation in voltage was strongest at higher voltages, whereas measurements were less consistent between mapping approaches in regions of low voltage. Thus, in regions of low voltage observed in AF with either bipolar mapping or OT, paired voltages during CSP diverged significantly more than in areas with higher voltage recordings. Despite this, the overall correlation between AF and CSP voltage at the highest measured voltages in AF was nonetheless weak. Bipolar voltage measured during CSP similarly correlated poorly with OT voltage in AF (supplemental figure 4)

**Supplemental Figure Legends**

**Supplemental figure 1A: Exclusion Criteria for Outlier EGM Recordings. The figure shows a histogram of EGM from four patients across different modalities and rhythms.**

A: Histogram of bipolar recordings collected during CSP, with the mean value indicated by a green line, the mean plus one standard deviation (SD) by a blue line, and the mean plus two SDs by an orange line. B: Histogram of CSP-OT recordings, with color-coding corresponding to the values from CSP-bipolar recordings. C: Histogram of bipolar recordings collected during atrial fibrillation (AF). D: Histogram of AF-OT recordings.

**Supplemental figure 1B. Closest point matching: The figure illustrates the point-matching technique.**

(A) Matching the closest point of CSP bipolar to CSP OT EGM. (B) Matching the closest point of CSP Bipolar to AF Bipolar EGM. (C) Matching the closest point of CSP OT to AF OT EGM. (D) Matching the closest point of AF Bipolar to AF OT EGM. The distance for all matches is 5 mm.

**Supplemental figure 2:** **The relationship between correlation coefficients (R-values) from peak-to-peak EGM recordings during CSP and AF and the mean voltages of the corresponding EGMs.**

Panels A and B show the correlation between R-values for CSP-Bipolar vs. CSP-OT and the mean voltages of CSP-Bipolar and CSP-OT, respectively. Panels C and D illustrate the correlation between R-values for AF-Bipolar vs. AF-OT and the mean voltages of AF-Bipolar and AF-OT, respectively. Panels E and F depict the correlation between CSP-Bipolar and AF-Bipolar R-values and the mean voltages of CSP-Bipolar and CSP-OT, respectively. Similarly, panels G and H present the correlation between CSP-OT and AF-OT R-values and the mean voltages of AF-Bipolar and AF-OT, respectively.

**Supplemental figure 3A: Pointwise comparison between EGMs recordings at different rhythms and conduction velocity**

The color-coded bars represent correlation values, where red denotes comparisons between bipolar recordings and CV-bipolar, blue corresponds to OT recordings and CV-OT. (A) Correlation between peak-to-peak voltage recordings and CV during CSP rhythm. (B) Correlation between GPMI voltage recordings and CV during CSP rhythm. (C) Correlation between peak-to-peak voltage recordings during AF and CV. (D) Correlation between GPMI voltage recordings during AF and CV.

**Supplemental figure 3B**: **Regional analysis of the relationship between bipolar and OT recordings during CSP and AF**

Each plot presents the results of linear regression analysis comparing the mean voltage in specific anatomical regions of the LA across 14 regions for each modality. (A) Linear regression of CV-bipolar versus CSP-bipolar recordings. (B) Linear regression of CV-OT versus CSP-OT recordings. (C) Linear regression of CV-bipolar versus AF-bipolar recordings. (D) Linear regression of CV-OT versus AF-OT recordings.

**Supplemental figure 4: Regional analysis of the relationship between bipolar and OT recordings during CSP and AF**

Each plot presents the results of linear regression analysis comparing the mean voltage in specific anatomical regions of the LA across 14 predefined regions for each modality. (A) Linear regression of CSP-OT versus AF-bipolar recordings. (B) Linear regression of CSP Bipolar versus AF OT recordings.

**Supplemental figure 1A**

**
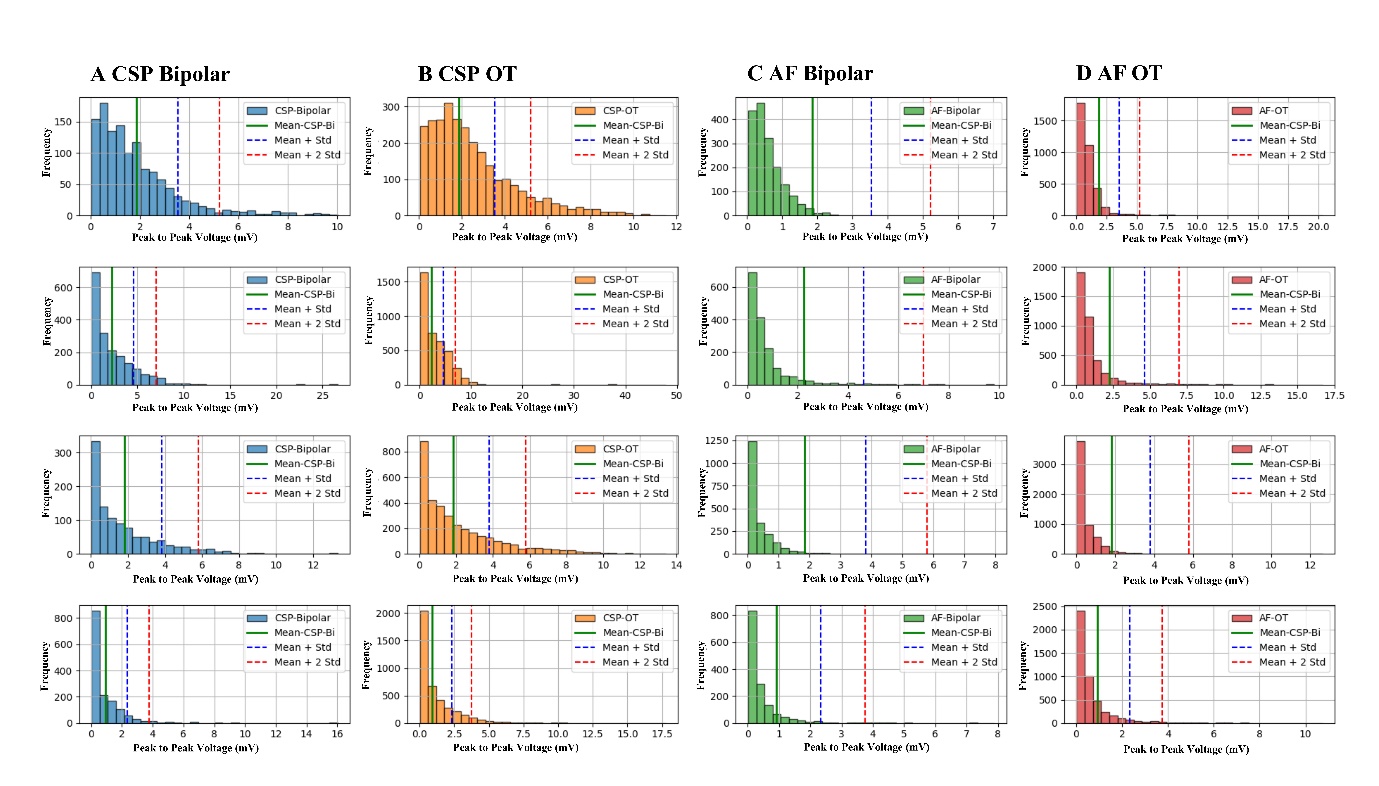
**

**Supplemental figure 1B**

**
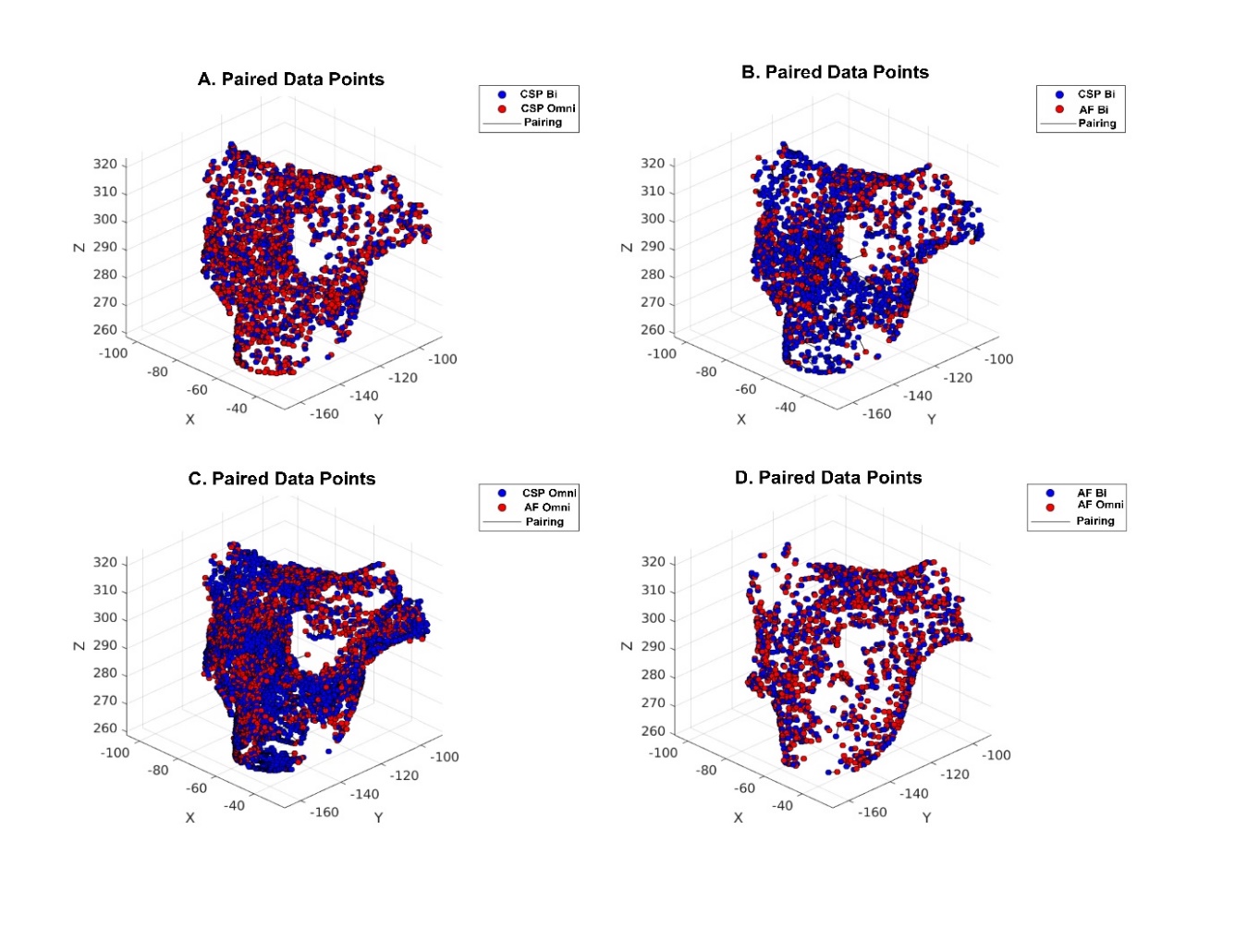
**

**Supplemental figure 2:**
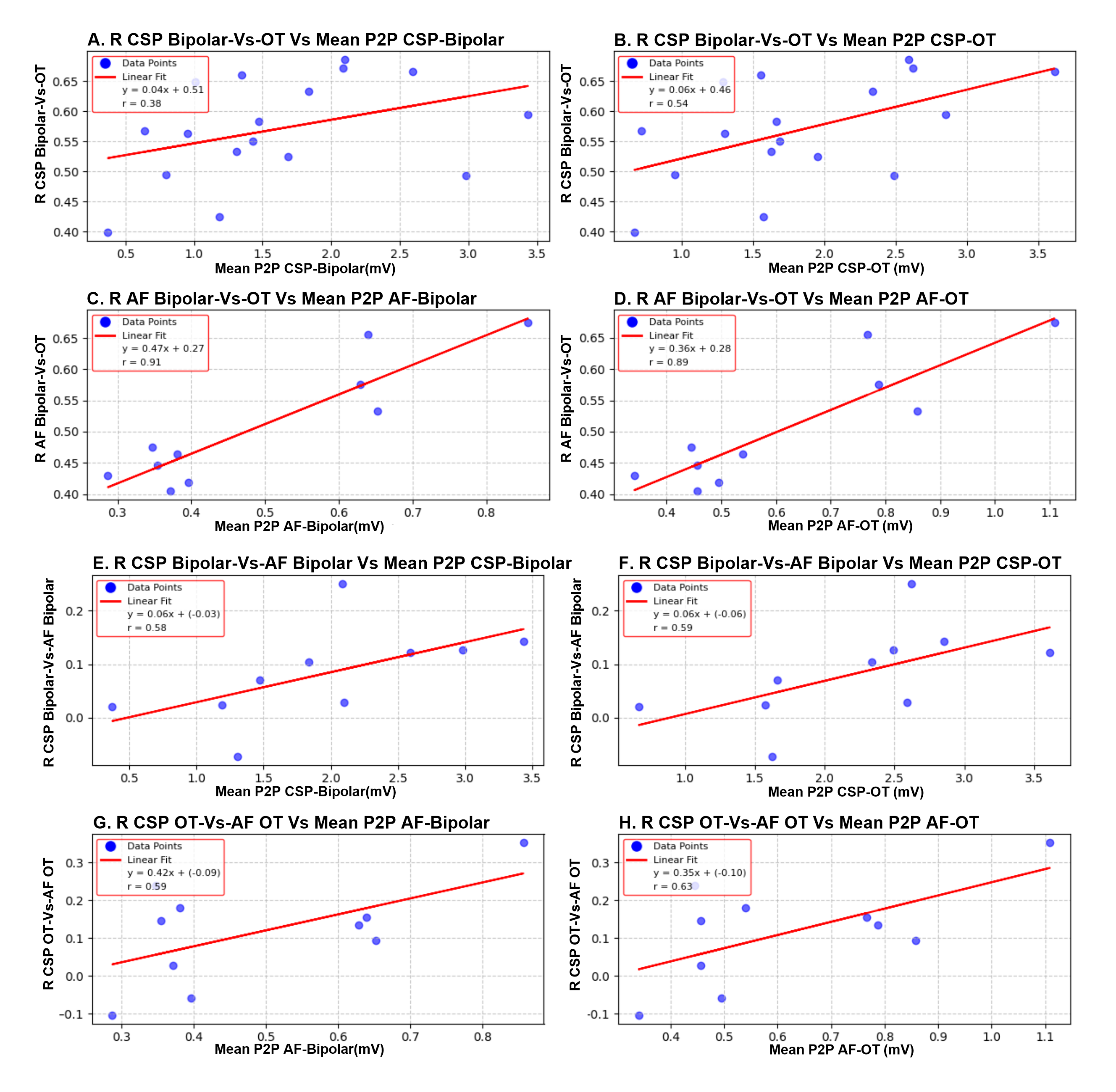


**
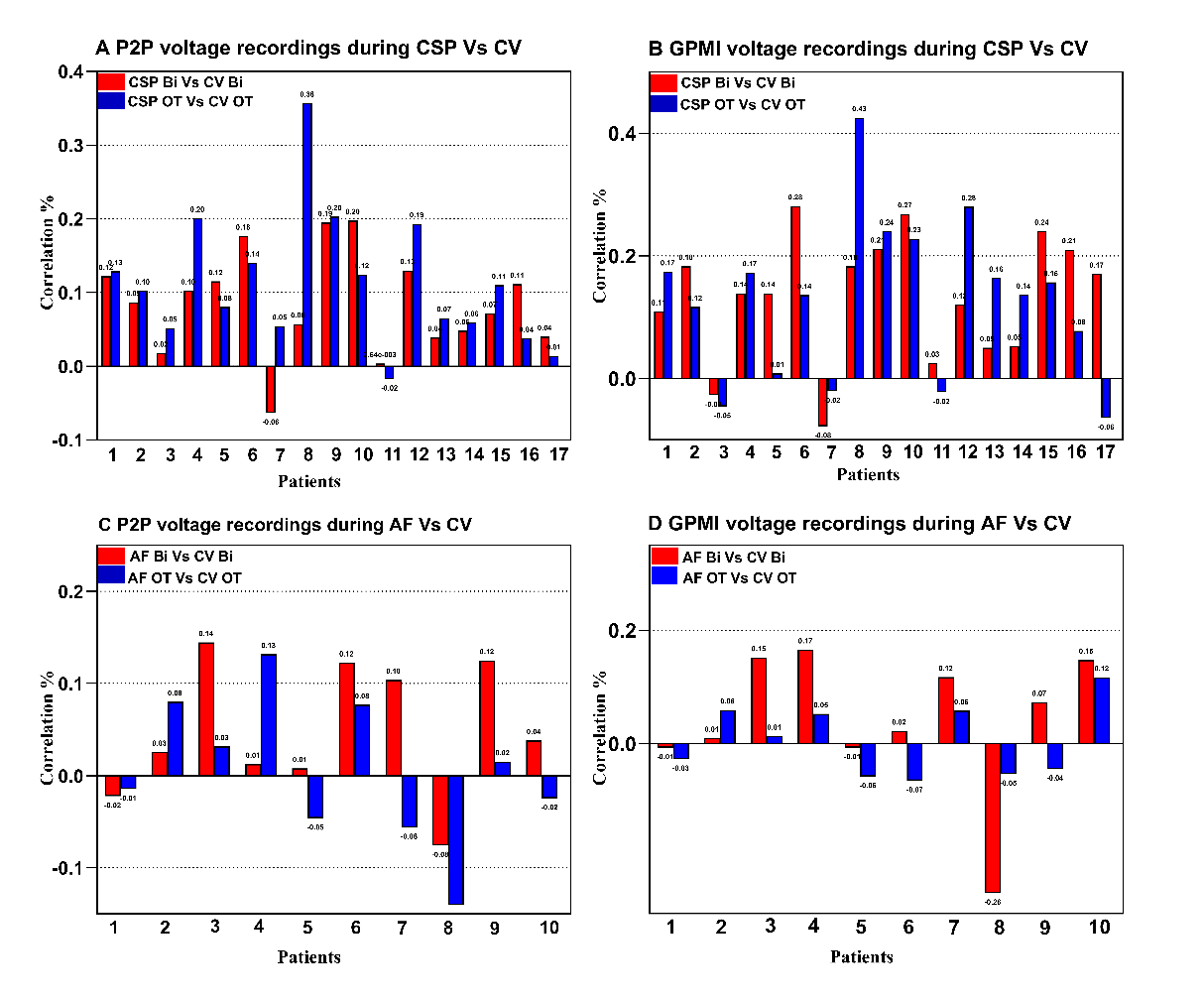
 Supplemental figure 3A**


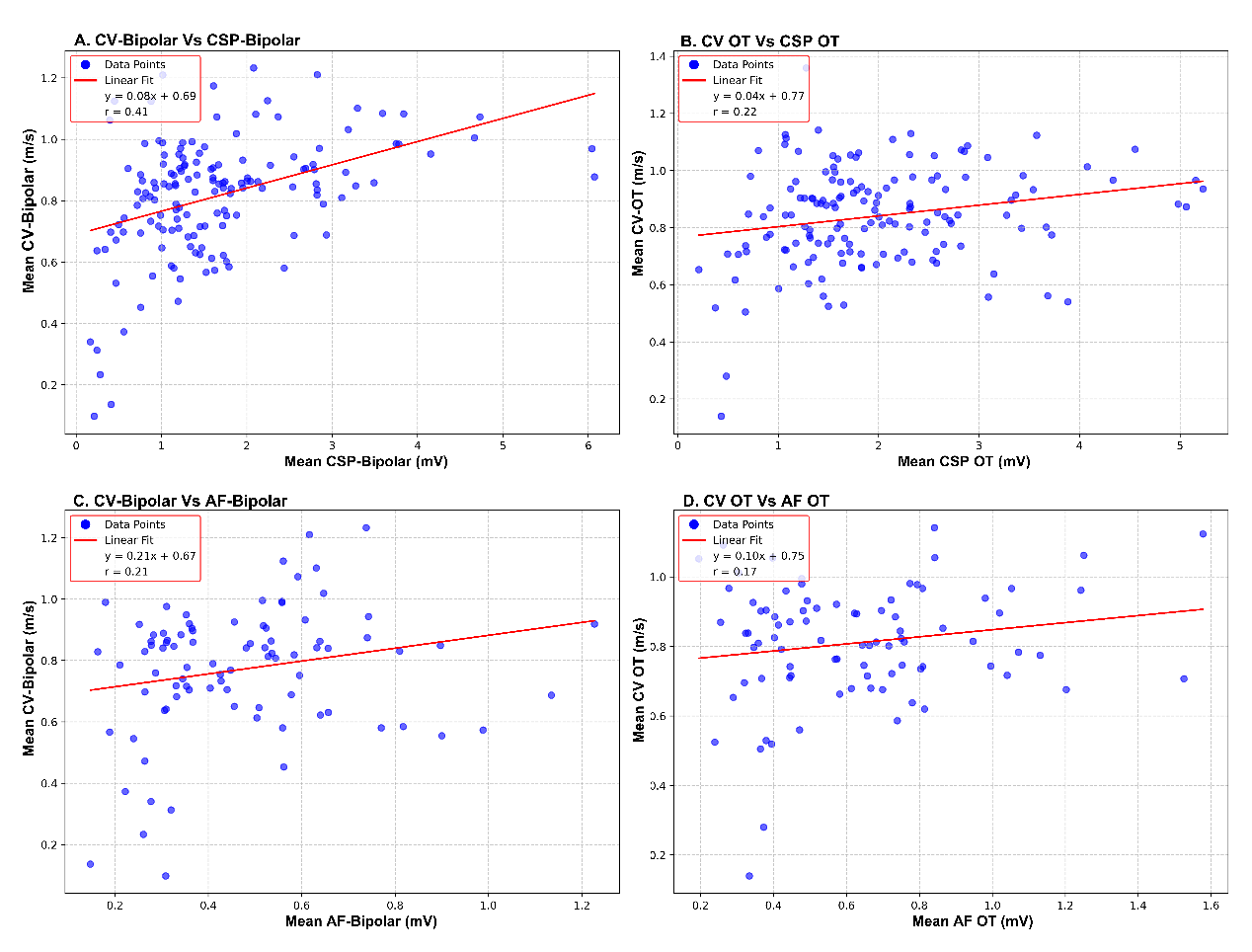
**Supplemental figure 3B**

**Supplemental figure 4**

**
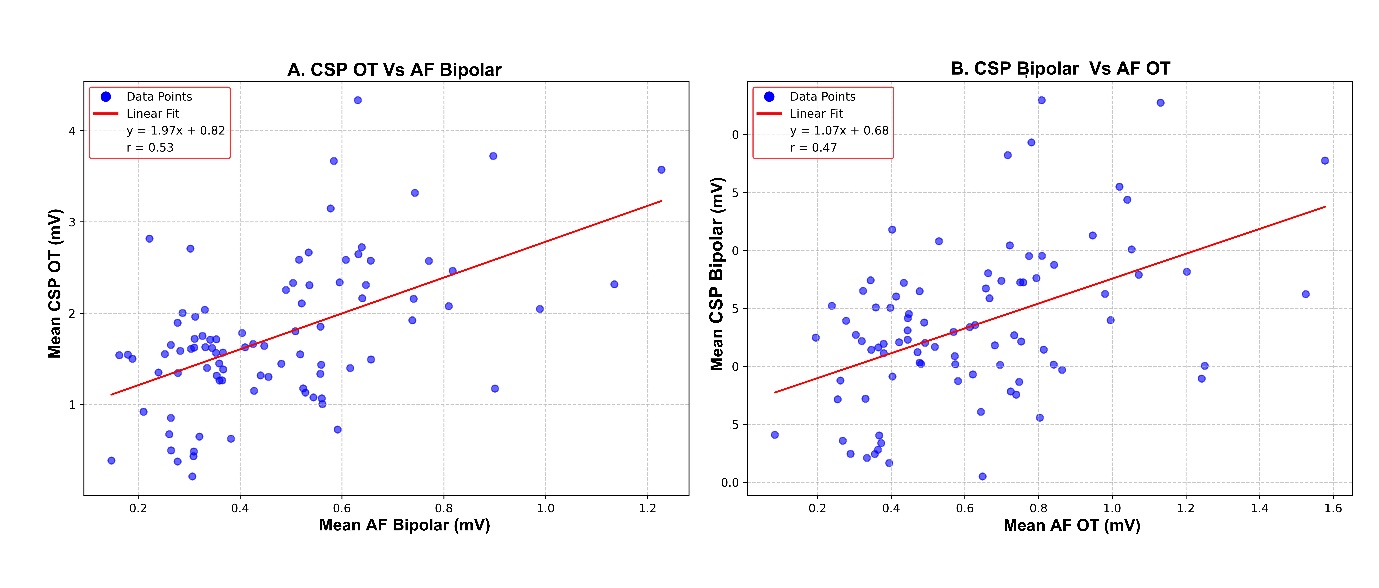
**

**Supplemental References**

1. Coveney S., Cantwell C., Roney C. Atrial conduction velocity mapping: clinical tools, algorithms and approaches for understanding the arrhythmogenic substrate. *Med Biol Eng Comput* 2022;60(9):2463–78. Doi: 10.1007/S11517-022-02621-0.

2. Coveney S., Corrado C., Roney CH., et al. Gaussian process manifold interpolation for probabilistic atrial activation maps and uncertain conduction velocity. *Philos Trans A Math Phys Eng Sci* 2020;378(2173). Doi: 10.1098/RSTA.2019.0345.

3. Roney CH., Whitaker J., Sim I., et al. A technique for measuring anisotropy in atrial conduction to estimate conduction velocity and atrial fibre direction. *Comput Biol Med* 2019;104:278–90. Doi: 10.1016/J.COMPBIOMED.2018.10.019.

4. Rodríguez-Mañero M., Valderrábano M., Baluja A., et al. Validating Left Atrial Low Voltage Areas During Atrial Fibrillation and Atrial Flutter Using Multielectrode Automated Electroanatomic Mapping. *JACC Clin Electrophysiol* 2018;4(12):1541–52. Doi: 10.1016/J.JACEP.2018.08.015.
